# Supplementary material for: A darkening spring: How preexisting distrust shaped COVID-19 skepticism
Source: PLoS One. 2022 Jan 26;17(1):e0263191. doi: 10.1371/journal.pone.0263191 (PMC8791533; doi:10.1371/journal.pone.0263191)
Supplement: S1 File — (PDF) [file pone.0263191.s001.pdf]

Supplemental Online Materials

Analytic Plan

We analyzed the survey data with Bayesian mixed-effects ordinal regression using the brms R package (1). We allowed for heterogeneity in both the intercepts for subject and item on the COVID-19 severity scale (2). We analyzed the data using a Bayesian framework because we were interested in the location and spread of the distribution of plausible estimated effects, rather than in the statistical significance of point estimates. In lieu of significance testing, we report the 95% credible interval of the posterior density of the coefficient estimates from our regression models.

Our analytic plan for modeling COVID-19 attitudes proceeded in several steps. First, we fit separate, maximal (or full) models to prewave and first-wave attitudes by predicting responses on the COVID-19 severity scale, using each participant's mean responses on all belief measures, as well as their political stance towards social issues. Second, we performed model comparison using leave-one-out cross validation criteria (3) to return the most parsimonious set of predictors that best predicts the data. We interpret this model as the best model of COVID-19 skepticism at the relevant time point. Degree of coherence between beliefs can be assessed by how well they correlate with one another; therefore, we also performed exploratory analyses of partial correlations of the predictors to illuminate finer-grained connections within the belief system. We also generated visualizations of how the predictors interface with political ideology.

Data and Analysis Scripts Availability

Survey data and R scripts for reproducing the reported analyses and figures are available open access at the Open Science Framework: https://osf.io/8xerq/?view_only=df620c3e86984668ae1a225028b5cd9b

Survey Materials

COVID-19 educational information:

Participants will first read a short paragraph describing COVID-19, which is adopted from the Wisconsin Department of Health Service's website.

> Coronaviruses are a large family of viruses that are common in both humans and animals. There are currently seven strains of human coronaviruses that have been identified. These common human coronaviruses typically cause a mild to moderate respiratory illness. Sometimes, new coronaviruses emerge.
>
> In 2019, a new strain of human coronavirus emerged, COVID-19. Illnesses associated with this virus were first reported in Wuhan, China, in December 2019.
>
> The main way COVID-19 is spread to others is when an infected person coughs or sneezes. This is similar to how influenza is spread. The virus is found in droplets from the throat and nose. When someone coughs or sneezes, other people near them can breathe in those droplets. The virus can also spread when someone touches an object with the virus on it. If that person touches their mouth, face, or eyes the virus can make them sick.
>
> In response to the number of people infected globally by COVID-19, as of March 11th the World Health Organization declared coronavirus a pandemic. Currently, there is no COVID-19 vaccine, but scientists are working on it.

Prewave Scales

COVID-19 Severity

1. COVID-19, commonly referred to as coronavirus, is no more severe than the flu.
2. I am afraid of dying from or contracting coronavirus. ***R***
3. Diseases that primarily affect the elderly are not that big of a deal.
4. COVID-19 is so rare there is no need for me to worry about it.
5. COVID-19 is the biggest threat to public health in recent years. ***R***

Foreign Threat

1. I am extra cautious around Asian people in order to protect against COVID-19.
2. One of the best ways to reduce the spread of COVID-19 is to stop immigration into the United States.
3. Because of COVID-19, America should reduce its interactions with China.
4. I find it racist when people refer to coronavirus as "Chinese coronavirus" or "Wuhan disease." ***R***

COVID-19 Origins
1. The global spread of COVID-19 was planned and orchestrated.
2. COVID-19 emerged from natural conditions. ***R***
3. COVID-19 was engineered in a laboratory.
4. The scientific community is spreading fake news about COVID-19.

Democrat Distrust
1. Some politicians are making a big deal out of COVID-19 for political gain.
2. COVID-19 is not as serious as some politicians say it is.
3. Politicians who downplay COVID-19's health risks are putting people's lives in danger. ***R***

Media Distrust
1. Some reporters and members of the media are making COVID-19 seem like a bigger deal than it really is.
2. News outlets are doing a good job communicating information relevant to COVID-19. ***R***
3. The media is politically motivated to play-up coronavirus to make Donald Trump and other Republicans look bad.

COVID-19 Vaccine Fears
1. I fear the government will use COVID-19 as an excuse to mandate vaccinations.
2. I am thankful that a COVID-19 vaccine does not exist.
3. A COVID-19 vaccine will be one of the most effective measures in reducing the spread of the virus. ***R***

Medical Distrust
1. Medical organizations like the CDC and WHO are untrustworthy.
2. I am skeptical of information provided by doctors and scientists.
3. The CDC and WHO strive to make recommendations in the public's best interest. ***R***
4. It is important to follow medical recommendations provided by the CDC and WHO.

First-wave Scales

COVID-19 Severity
1. COVID-19, commonly referred to as coronavirus, is no more severe than the flu.
2. I am afraid of dying from or contracting coronavirus. ***R***
3. Diseases that primarily affect the elderly are not that big of a deal.
4. COVID-19 is so rare there is no need for me to worry about it.
5. COVID-19 is the biggest threat to public health in recent years. ***R***

Foreign Threat

1. I am extra cautious around Asian people in order to protect against COVID-19.
2. One of the best ways to reduce the spread of COVID-19 is to stop immigration into the United States.
3. Because of COVID-19, America should reduce its interactions with China.
4. I find it racist when people refer to coronavirus as “Chinese coronavirus” or “Wuhan disease.” ***R***

COVID-19 Origins

1. The global spread of COVID-19 was planned and orchestrated.
2. COVID-19 emerged from natural conditions. ***R***
3. COVID-19 was engineered in a laboratory.
4. The scientific community is spreading fake news about COVID-19.

Democrat Distrust

1. Some politicians are making a big deal out of COVID-19 for political gain.
2. COVID-19 is not as serious as some politicians say it is.
3. Politicians who downplay COVID-19’s health risks are putting people’s lives in danger. ***R***

COVID-19 Vaccine Fears

1. I fear the government will use COVID-19 as an excuse to mandate vaccinations.
2. I am thankful that a COVID-19 vaccine does not exist.
3. An effective coronavirus vaccine will save many lives. ***R***

Medical Organization Distrust

1. Medical organizations like the CDC and WHO are untrustworthy.
2. I am skeptical of information provided by doctors and scientists.
3. The CDC and WHO strive to make recommendations in the public’s best interest. ***R*** 4. It is important to follow medical recommendations provided by the CDC and WHO.

General Vaccine effectiveness

1. Your chances of getting a disease after being vaccinated against it are incredibly low. ***R***
2. Improved nutrition and sanitation played a larger role than vaccines in the decline of diseases like measles.
3. Recent outbreaks of diseases like measles and whooping cough show that vaccines don’t work very well.
4. Vaccines are one of the most effective medical treatments. ***R***

Opening up the economy

1. The response to stop the spread of COVID-19 has caused more harm than the virus itself.
2. We should stop social distancing as soon as possible to kickstart the economy.
3. We should reopen the economy once we are confident there will be no more serious coronavirus outbreaks. ***R***

R = reverse coded

Prior Specification

Bayesian ordinal modeling requires placing prior distributions in the model describing our prior expectation of the cumulative likelihood that a given Likert response on the dependent variable (here, response on the COVID-19 severity scale) will be chosen. We used Gaussian priors centered at the cumulative proportion of responses for each item, with a standard deviation of .25. These priors do not bias the results of our analysis in any way, and are useful in increasing the efficiency of the Markov-chain Monte Carlo (MCMC) algorithm used to estimate the posterior distributions of regressors, which express a probability distribution over likely effect sizes for each regressor in the linear model. Here, we reported effects as beta estimates from the full model, with their associated 95% credible intervals, which communicates the range of beta estimates within the interior 95% of the posterior distribution. To protect against model overfitting, we placed regularizing priors on the model predictors to bias parameter estimates towards more conservative values (i.e., effects closer to zero (4)).

Leave-one-out Cross Validation

We performed leave-one-out (loo) cross validation (3) on multiple models with subsets of the predictors using the loo_compare method in the brms R package. This approach allowed us to uncover the model with the smallest set of predictors with the highest predictive accuracy. Leave-one-out cross validation measures the predictive accuracy of a model by using all data minus one sample as a training dataset (which is used to estimate the model's parameters), and then using

that fitted model to predict the value of the held-out sample. This procedure is repeated for all samples in the dataset, and the predictive accuracy across all samples is then aggregated to form an estimate of how well the model predicts unseen data. The loo_compare method estimates the difference in the predictive accuracy between a set of models and can therefore be used to gauge the relative predictive accuracy of the models in the set.

Our goal is to find the most parsimonious model that best predicts the data (i.e., the model with the smallest set of predictors with an estimated predictive accuracy within the standard error of the best-predicting model). We interpret this parsimonious model in relation to the partial correlations of the predictors to generate an explanatory account of the factors shaping COVID-19 skepticism prior to and during the first wave of cases. To further explore how these predictors interface with participants' political ideologies, we generated visualizations showing how responses on the belief measures varied as a function of the participant's response on the politics scale. We also computed partial correlations between the predictors and political stance regarding social issues. Participants reported their social and political attitudes on a 7-point Likert scale, with choices ranging from "Very Liberal" to "Very Conservative".

SOM References

1. Bürkner P-C. brms : An R Package for Bayesian Multilevel Models Using Stan. J Stat Soft [Internet]. 201;80(1). Available from: http://www.jstatsoft.org/v80/i01/

2. Barr DJ, Levy R, Scheepers C, Tily HJ. Random effects structure for confirmatory hypothesis testing: Keep it maximal. Journal of Memory and Language. 2013 Apr;68(3):255–78.

3. Vehtari A, Gelman A, Gabry J. Practical Bayesian model evaluation using leave-one-out cross-validation and WAIC. Stat Comput. 2017 Sep 1;27(5):1413–32.

4. McElreath R. Statistical Rethinking [Internet]. Oreilly.
