# Supplementary material for: A darkening spring: How preexisting distrust shaped COVID-19 skepticism
Source: PLoS One. 2022 Jan 26;17(1):e0263191. doi: 10.1371/journal.pone.0263191 (PMC8791533; doi:10.1371/journal.pone.0263191)
Supplement: Attachment — Submitted filename: Response to Reviewers.docx [file pone.0263191.s002.docx]

**Response to Reviewers**

Hunter Priniski	Keith Holyoak

We thank the reviewers for their careful comments and address each point below.

**Journal Requirements**

1. Please ensure that your manuscript meets PLOS ONE's style requirements, including those for file naming. The PLOS ONE style templates can be found at
   <https://journals.plos.org/plosone/s/file?id=wjVg/PLOSOne_formatting_sample_main_body.pdf> and 
   <https://journals.plos.org/plosone/s/file?id=ba62/PLOSOne_formatting_sample_title_authors_affiliations.pdf>

Completed

2. Please provide additional details regarding participant consent. In the ethics statement in the Methods and online submission information, please ensure that you have specified what type you obtained (for instance, written or verbal, and if verbal, how it was documented and witnessed). If your study included minors, state whether you obtained consent from parents or guardians. If the need for consent was waived by the ethics committee, please include this information.

We added the following *Ethics Statement* subsection to our Methods section:

*Ethics Statement*

The Institutional Review Board of the University of California, Los Angeles, approved our study, and written consent was received for each participant. All participants were over 18 years of age.

Preparation of this paper was supported by NSF Grant BCS-1827374.

Please state what role the funders took in the study: The funders had no role in study design, data collection and analysis, decision to publish, or preparation of the manuscript.

This info was added to our Acknowledgements section, which now reads:

The funders of this study had no role in study design, data collection and analysis, decision to publish, or preparation of the manuscript.

4. Thank you for stating the following in the Acknowledgments Section of your manuscript:

Preparation of this paper was supported by NSF Grant BCS-1827374.

Please note that funding information should not appear in the Acknowledgments section or other areas of your manuscript. We will only publish funding information present in the Funding Statement section of the online submission form. 
Please remove any funding-related text from the manuscript and let us know how you would like to update your Funding Statement. Currently, your Funding Statement reads as follows:

Preparation of this paper was supported by NSF Grant BCS-1827374.

Please include your amended statements within your cover letter; we will change the online submission form on your behalf.

Funding details have been removed: see revised Acknowledgement above.

5. We note that you have stated that you will provide repository information for your data at acceptance. Should your manuscript be accepted for publication, we will hold it until you provide the relevant accession numbers or DOIs necessary to access your data. If you wish to make changes to your Data Availability statement, please describe these changes in your cover letter and we will update your Data Availability statement to reflect the information you provide.

Our data and analysis scripts are hosted publicly on the Open Science Framework. As one reviewer seems to have a tough time locating these files, we included a link to them in our Results section.

6. Please include your full ethics statement in the ‘Methods’ section of your manuscript file. In your statement, please include the full name of the IRB or ethics committee who approved or waived your study, as well as whether or not you obtained informed written or verbal consent. If consent was waived for your study, please include this information in your statement as well.

We added an Ethics subsection to the Materials. See response to point 2 above.

7.  Please review your reference list to ensure that it is complete and correct. If you have cited papers that have been retracted, please include the rationale for doing so in the manuscript text, or remove these references and replace them with relevant current references. Any changes to the reference list should be mentioned in the rebuttal letter that accompanies your revised manuscript. If you need to cite a retracted article, indicate the article’s retracted status in the References list and also include a citation and full reference for the retraction notice.

We did some small editing to the reference list for style. No references were added or removed.

**Reviewer Comments**

Reviewer #1:

We thank Reviewer #1 for their careful read of the manuscript. We individually address each point they raised below.

1. clarify the number of scales used in pre-wave (6 in text, 7 in table)

We reported the wrong number in the original text. We updated the numbers, so they match.

2. recheck the numbering of figures and their reference in text (figure 1 appears twice)

We updated the figure counts.

3. recheck the interpretation of the skewness (left, right) for democrats vs. republicans for all distributions

We made these changes in the manuscript. And thank the reviewer for their careful eye.

4. check for multicollinearity prior to presenting CI

The predictors will be strongly correlated by design as correlations between predictors is necessary to measure coherence. We thank the reviewer for raising concerns about the potential for a high degree of collinearity between the predictors which may undermine results. In Bayesian linear models, collinearity between predictors results in flat (uninformative) posteriors estimates, even if one of the predictors in fact is predictive. More information about Multicollinearity in posterior distributions of Bayesian multiple regression models can be found in R. McElreath’s textbook on Bayesian Modeling *Statistical Rethinking* (pp. 141-142).

As we saw in the full model, the posteriors of the predictors are not flat but rather informative, therefore alleviating concerns of collinearity between predictors. We added a justification for this in the manuscript before reporting the results.

5. recheck the interpretation of correlation coefficients (r=.43 is moderate, not strong)

We updated our interpretations of the correlation coefficients such that they mirror conventional nomenclature.

6. consistent use of the same variable names in each figure/table/ text (eg. democrat distrust and democrat hoax, political attitude and conservative politics). If they refer to different variables, please explain the differences.

The variable names were updated in both Figure 4 and 10, where we used conservative politics as a label for Political Attitudes. We changed both labels to Political Attitudes. We also fixed the labeling error in Figure 10 where we used Democratic Hoax rather than Democrat Distrust. This was fixed as well.

Overall, this paper can be viewed as a good presentation of the insights of the skeptical attitude regarding covid.

**Reviewer #2:** no suggestions for revision

The contribution of the current paper is not sufficient for publication in this journal. Thus, I have decided to reject the paper "A Darkening Spring: How Preexisting Distrust Shaped COVID-19 Skepticism".

**Reviewer #3:**

Solid work. Would have liked additional comments on the development of the framework and specifics to address potential new narratives as to inform better policy that takes into account the insights provided for future issues.

We added a suggestion in the discussion regarding how we believe a coherence mechanism will also be at play in future pandemics. Therefore, large medical organizations should be prompt in addressing these coherence-based connections between science and distrust.

**Reviewer #4:** no suggestions for revision
